# Role of Artificial Intelligence in the Diagnosis and Management of Pulmonary Embolism: A Comprehensive Review

**DOI:** 10.3390/diagnostics15070889

**Published:** 2025-04-01

**Authors:** Ahmad Moayad Naser, Rhea Vyas, Ahmed Ashraf Morgan, Abdul Mukhtadir Kalaiger, Amrin Kharawala, Sanjana Nagraj, Raksheeth Agarwal, Maisha Maliha, Shaunak Mangeshkar, Nikita Singh, Vikyath Satish, Sheetal Mathai, Leonidas Palaiodimos, Robert T. Faillace

**Affiliations:** 1 Department of Medicine, New York City Health + Hospitals/Jacobi, Albert Einstein College of Medicine, New York, NY 10461, USA; nasera1@nychhc.org (A.M.N.); vyasr1@nychhc.org (R.V.); morgana2@nychhc.org (A.A.M.); agarwalr1@nychhc.org (R.A.); maliham@nychhc.org (M.M.); mangeshs@nychhc.org (S.M.); singhn33@nychhc.org (N.S.); satishv@nychhc.org (V.S.); leonidas.palaiodimos@nychhc.org (L.P.); robert.faillace@nychhc.org (R.T.F.); 2 Department of Medicine, Montefiore Wakefield Medical Center, New York, NY 10461, USA; mukhtadirmaaz@gmail.com; 3 Department of Cardiology, University of Nebraska Medical Center, Omaha, NE 68198, USA; 4 Department of Cardiology, Montefiore Medical Center, Albert Einstein College of Medicine, New York, NY 10461, USA; sanjana94nagraj@gmail.com (S.N.);

**Keywords:** artificial intelligence, artificial neural networks, deep convolutional neural networks (DCNN), machine learning, natural language processing (NLP), pulmonary embolism

## Abstract

Pulmonary embolism (PE) remains a critical condition with significant mortality and morbidity, necessitating timely detection and intervention to improve patient outcomes. This review examines the evolving role of artificial intelligence (AI) in PE management. Two primary AI-driven models that are currently being explored are deep convolutional neural networks (DCNNs) for enhanced image-based detection and natural language processing (NLP) for improved risk stratification using electronic health records. A major advancement in this field was the FDA approval of the Aidoc© AI model, which has demonstrated high specificity and negative predictive value in PE diagnosis from imaging scans. Additionally, AI is being explored for optimizing anticoagulation strategies and predicting PE recurrence risk. While further large-scale studies are needed to fully establish AI’s role in clinical practice, its integration holds significant potential to enhance diagnostic accuracy and overall patient management.

## 1. Introduction

Venous thromboembolism (VTE), which includes deep vein thrombosis (DVT) and pulmonary embolism (PE), affects up to 600,000 patients annually in the United States (US) [1] and has a significant socioeconomic impact with an estimated annual healthcare cost between USD 13.5 billion and USD 27.2 billion [2]. It leads to an estimated mortality of 10–30% within 30 days, predominantly contributed to by patients with PE [1]. Furthermore, it contributes to increased morbidity in these patients due to the development of chronic conditions like chronic thromboembolic pulmonary hypertension [1,3]. Although there has been a reduction in the in-hospital case-fatality rate of stable patients with PE in the US between 1999 and 2008, the fatality rate is 6.1% in stable patients, rising up to 35.2% for unstable patients [4]. Furthermore, data from a Danish population-based registry indicate that 30 years after the first PE, the adjusted mortality rate ratio is 2.77 compared to the general population when adjusted for baseline characteristics, cancer, major cardiopulmonary diseases, and chronic conditions, indicating a long-term impact of PE, which is traditionally considered an acute condition [5].

Considering the health and economic impact of PE, timely diagnosis and management are incredibly important. Current diagnostic methods involve scoring systems to risk stratify patients and the use of imaging, such as computed tomography pulmonary angiogram (CTPA), for thrombus detection. However, despite being the gold standard test for diagnosing PE, CTPA can be subject to errors caused by contrast opacification issues, motion artifacts, increased body habitus, and inter-reader variability [6,7]. Prophylaxis and treatment, such as with anticoagulation, is also not without risk, and decision-making regarding initiation and discontinuation can be complex. Given these challenges, there is an opportunity to incorporate automation and artificial intelligence (AI) into clinical practice to improve the identification of at-risk patients, diagnosis, and management of PE. As an example demonstrating its utility, Ayobi et al. conducted a retrospective study using an AI algorithm, CINA-PE, to assess CTPA scans for aiding PE diagnosis [8]. They found that it was capable of identifying 76% of missed PEs and had a per-case sensitivity of 93.9% and specificity of 94.8%. Through this review, we aim to explore the current scope of AI in PE and synthesize data regarding its applications in risk detection, prevention, diagnosis, and management.

## 2. What Is Artificial Intelligence?

AI is defined as computer systems that demonstrate or replicate a particular facet of human intelligence or behavior, including activities like learning, reasoning, and problem-solving [9]. It is a non-classical computer programming method that encompasses machine learning (ML), deep learning (DL), and artificial neural networks (ANNs) [10].

Deep learning using ANNs is an AI method inspired by the human brain’s neuronal function. In an ANN, the input layer collects data features and transmits them with varying weights to neurons in hidden layers. Each hidden neuron aggregates the weighted information, applies additional weighting, and forwards it to the next layer. This process facilitates gradual feature extraction and transformation across the network’s layers. The final output layer provides the answer the ANN was trained for. During training, backpropagation adjusts the neuron weights to optimize the network’s performance. However, this can also lead to false associations due to biases in the training data [11]. To conceptualize this, ANNs can be thought of as the machines equivalent of neurons, inspired by the neurons in the human brain. ANNs encompass various subtypes, including deep convoluted neural networks (DCNNs), which are particularly effective for image-processing tasks.

Techniques like natural language processing (NLP) use neural network architectures to process text data. NLP converts text data into machine-readable code, but early versions had limited use in healthcare due to complexities in medical terminology [12]. Advancements in machine learning and neural networks have allowed neural NLP to extract data accurately and produce human-like text. By applying neural NLP to free-text medical records, vast amounts of information can be accessed to potentially aid diagnosis [13].

Neural NLPs use neural networks and text language from the internet to extract and produce unscripted text language accurately, similar to the tasks carried out by the human brain [14]. Combining this process with free text data available from medical records can help with pattern recognition and facilitate the diagnosis of certain conditions. Additionally, this method can help advance research through automation, where large amounts of data can be processed and extracted in a fraction of the time [13].

**Figure 1:**
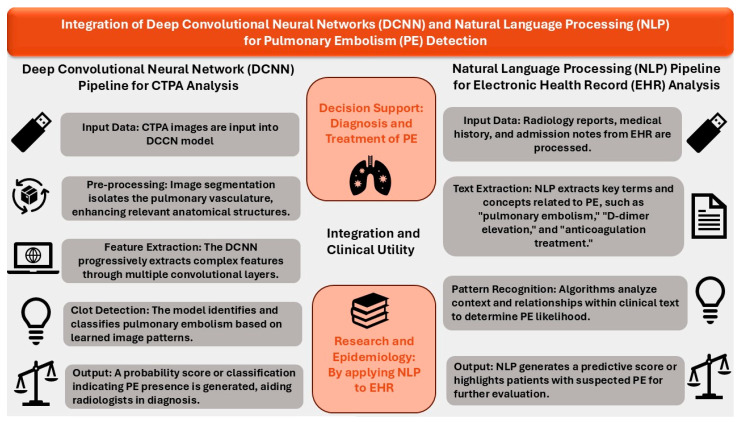
The integration of DCNN and NLP for PE detection.

With increasing access to vast data and advanced computing power, complex neural networks can be developed to uncover previously overlooked associations and risks, enhancing the ability to deliver optimal healthcare in collaboration with AI [11,15]. Figure 1 represents the integration of DCNN and NLP in the diagnosis of PE.

**Figure 2:**
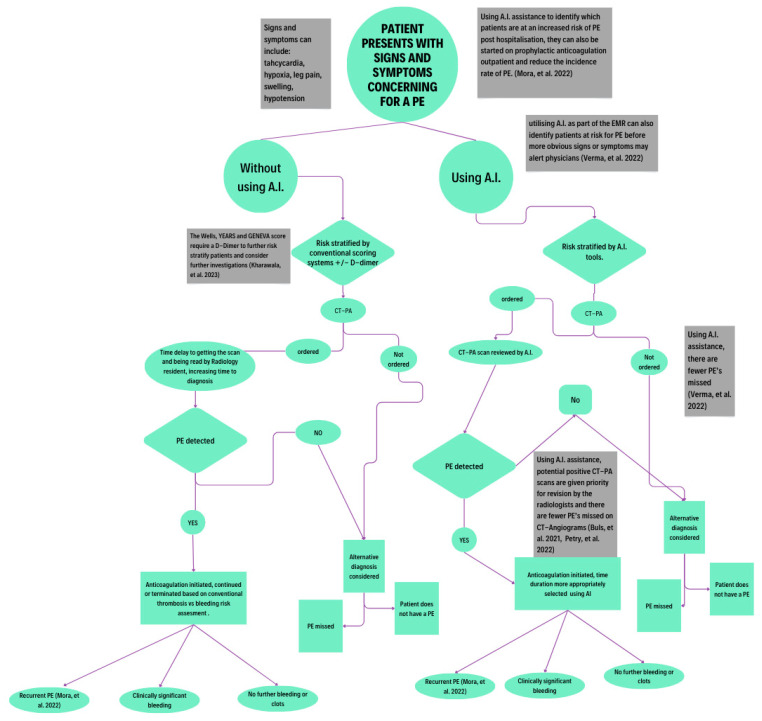
Work-flow example of clinical utilization of AI in diagnosis and management of PE [16,17,18,19,20,21,22,23].

In the following sections, we will discuss recent studies that have employed various forms of AI to better detect, diagnose, and treat PEs. This includes the role of DCNN in image processing to identify PE from CTPA images and utilization of NLP in risk-stratifying patients using electronic health records. Moreover, we will discuss the current literature that delves into utilizing AI for PE management in various clinical settings. Figure 2 highlights the key areas where AI can be safely incorporated for improving clinical efficiency in the diagnosis and management of PE.

## 3. AI Models Using Convolutional Neural Networks (CNNs)

Deep convolutional neural networks (DCNNs) are models designed to process and analyze data, primarily from images, with the ability to identify patterns and extract features. This allows them to be applied to radiologic imaging, potentially enhancing the diagnostic accuracy of PEs on CTPA [16,17,18,19]. A DCNN model can be trained on labeled CTPA images, using preprocessing steps to isolate pulmonary vasculature and progressively identify increasingly complex features, such as clots, through layered feature extraction [24].

### 3.1. Enhanced CTPA-Based Diagnosis of PE Using FDA-Approved AIDOC Models

Aidoc© (Tel Aviv, Israel), which is an AI-based company, recently launched their AI-based models to aid in the diagnosis of PE. This FDA-approved AI model developed by Aidoc©, (Tel Aviv, Israel) Medical has paved the way for successfully incorporating AI in aiding physicians who are managing patients with PE [16,17,18,19] (Table 1). In this model, the reports for the computed tomography (CT) scan generated by the AI model were compared to previously generated radiologist reports. In the event of discrepancies, a panel adjudication was conducted to define the gold standard reference. The resultant findings demonstrate a strong diagnostic performance by the AI models, with high specificity and negative predictive value (NPV). However, it must be noted that the retrospective nature of these studies precluded a head-to-head comparison between AI models and radiologist reports. Additionally, only cases with discrepancies between AI reports and radiologist reports were reviewed by a panel of blinded experts. Table 1 summarizes the studies that evaluated this FDA-approved model developed by Aidoc© medical.

### 3.2. Alternate DCNN-Based Models for Improved CTPA-Based Diagnosis of PE

AI has been used to successfully detect PE from CTPA images using different models [16,17,18,19]. A meta-analysis of five retrospective studies evaluating the use of AI algorithms based on DCNNs in the detection of PE from CTPAs yielded a pooled sensitivity and specificity of 88% and 86%, respectively, with radiologist evaluation of images as a reference [24].

Weikert et al. demonstrated that their cloud-based prototype algorithm, which utilized CNN, reached a sensitivity of 92.7% and specificity of 95.5% in diagnosing PE on CTPAs [25]. This AI model was then successfully trained and validated on 28,000 CTPAs from nine medical centers utilizing 17 different models of CT scanners before external validation [25]. This is similar to the use of DCNN for detecting stroke and intracranial hemorrhage, which is now being successfully extrapolated to improving the diagnosis of PE [26,27].

The interpretation of CTPAs by radiologists can be limited by suboptimal image acquisition, either due to motion artifacts or poor contrast enhancement. Ebrahimian et al. compared 104 CTPA studies labeled by radiologists as suboptimal to 226 CTPA studies labeled as optimal and demonstrated that the performance of their AI model was not affected by suboptimal image acquisition. The studied AI model detected PE in both groups (suboptimal and optimal CTPAs) with high sensitivity and specificity in comparison to radiologists’ interpretation of suboptimal CTPA (AI model: sensitivity 100%; specificity 89%; AUC 0.89, 95% CI 0.80–0.98) and optimal CTPA (AI model: sensitivity 96%; specificity 92%; AUC 0.87, 95% CI 0.81–0.93) [28]. Although a prior study using AIDOC version 1.3, Tel Aviv, Israel AI algorithms by Buls et al. yielded a lower sensitivity of 73% for detecting PE compared to radiologist interpretation, the lower sensitivity was attributed to a higher prevalence of chronic PE and artifacts due to superimposed anatomy [16,28]. This demonstrates that the currently approved and validated AI models are not without pitfalls, which need further refinement in their current coding methods.

## 4. AI Models Using NLP

NLP can leverage information from radiology reports, the medical history, and admission notes, and by extracting text data relating to concepts such as PE, it can highlight patients that have a potential diagnosis [20,29,30,31]. Hence, NLP can harness the EHR to more easily assess large-scale hospital data, identifying patients with PE. A 2022 cross-sectional study among five hospitals in Canada demonstrated NLP’s ability to effectively identify PE by analyzing radiology reports, achieving a 0.9 positive predictive value and 0.96 area under receiver operator curve (AUROC) in a dataset of 1551 hospitalizations [20]. This highlights the potential benefit of integrating NLP with EHR systems, which can provide accurate data regarding PE incidence and rate for research and statistical reasons. It may also have the clinical benefit of identifying patients who have PE, thus leading to prompt anticoagulation treatment, and those who require further evaluation and risk assessment for prophylaxis.

However, the challenge arises in integrating NLP into complex EHR systems, with a focus on maintaining compliance with security and confidentiality requirements, such as the Health Insurance Portability and Accountability Act (HIPAA).

## 5. Role of AI in Enhanced Diagnosis and Management of PE

**Figure 3:**
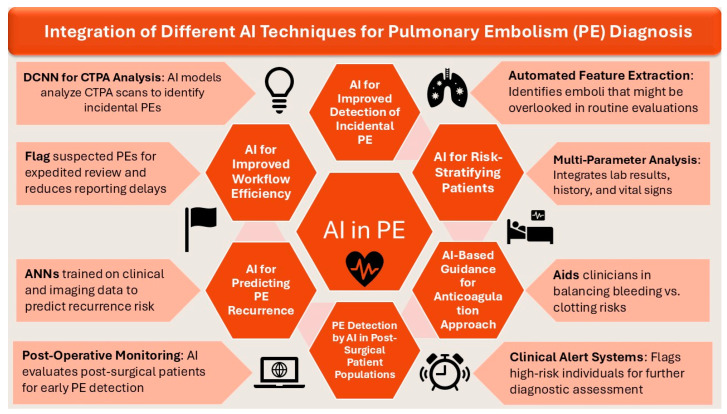
The integration of A.I. techniques for PE diagnosis.

Integration of various AI techniques, including NLP and DCNN, have resulted in improved risk stratification of patients and diagnosis of PE. Moreover, practical incorporation of AI techniques in this cohort of patients with PE have also helped in improving the approach to anticoagulation and predicting the recurrence rate of PE. Figure 3 consolidates the integration of AI in management of PE.

### 5.1. AI for Improved Detection of Incidental PE

Incidental PEs that are not the primary focus of the imaging study can be easily overlooked by radiologists. However, AI-based models have demonstrated enhanced sensitivity in detecting incidental PEs in contrast-enhanced chest computed tomography (CT) scans. Wiklund et al. demonstrated the efficiency of the AIDOC medical-based AI model in detecting 68 of 75 incidental PEs (iPEs) among a cohort of 1892 patients with different malignancies, whereas clinical radiologists only detected 16 of 75 iPEs [32]. Similarly, Langius-Wiffen et al. also reported an increased sensitivity in detecting iPEs in chest CTs when an AI-based model was utilized [18]. Although not all subsegmental PEs in the general population require treatment, patients with malignancies are at a higher risk of recurrent PE, underscoring the importance of accurate diagnosis of iPE. Patients with untreated iPEs can have a higher risk of recurrent PE, even if the initial PE had resolved [33]. The enhanced capability of AI can have significant clinical implications by potentially reducing the risk of missing iPE [21].

### 5.2. AI for Improved Workflow Efficiency

AI can streamline the PE diagnosis workflow by prioritizing cases with suspected PEs for radiologist review. Batra et al. deployed a unique AI-based worklist prioritization system wherein every CTPA was analyzed by an FDA-approved AI model [34]. Detected PE cases were subsequently prioritized to the top of radiologists’ worklists, resulting in substantially reduced waiting and turnaround times for reporting. Rothenberg et al. corroborated these findings, also observing shortened workflow times for radiologists utilizing AI assistance [35]. Such efficiency gains can have critical clinical implications, as demonstrated by Petry et al., who reported that implementing an AI notification and triage system significantly reduced the average patient length of stay from 7.91 to 5.83 days (*p* = 0.034) in patients with PE [21].

### 5.3. AI for Predicting PE Recurrence

PE has high recurrence rates, with 13–15% of patients experiencing recurrence after a first PE episode [36]. Existing prediction scores for recurrence are only applicable to unprovoked PEs [37,38,39]. To address this, Martins et al. used three ANN models using different sets of variables from 235 patients [36]. These models demonstrated high accuracy in classifying patients prone to recurrent PE. All models showed high predictive efficacy for recurrent PE, with an accuracy of 92.8%, 98.5%, and 97.1% for the three models. The findings suggest that these ANN models, once properly validated, could assist clinicians in making informed decisions about PE treatment by overcoming the restrictions of current methodologies.

### 5.4. AI for Risk-Stratifying Patients

There are many data points, such as laboratory results, presenting history, and vitals, that can contribute to a suggestive diagnosis of PE. AI may play a role in incorporating these numerous elements to assist with prediction of PE likelihood. A single-center retrospective study involving 917 patients evaluated the diagnostic accuracy of an AI-generated model by comparing it to established scoring systems for the diagnosis PE [22]. The study assessed 25 parameters, utilizing CTPA results as the gold standard. The AI model demonstrated a diagnostic accuracy of 84.6%, surpassing the predictive performance of the Wells score, YEARS score, and revised Geneva score. These findings highlighted the potential diagnostic capability of the AI-generated model over conventional scoring systems in the detection of PE, including the physician’s clinical acumen in the detection of PE.

Studies have shown that machine learning models are superior to conventional scoring systems at predicting PE risk using electronic health records (EHRs) [40,41]. Ma et al. implemented a two-stage hierarchical ML model for PE prediction where common risk factors for PE were compiled from multiple hospitals to construct an initial prediction model. This was followed by department-specific ML models in the second stage [42]. The study included a total of 9213 adult patients who were admitted under various departments, and PE was confirmed in 1165 of them. Variables used in the first stage model (total *n* = 70) included catheters and duration, D-dimer, prothrombin time (PT), and activated partial thromboplastin time (aPTT). Additional department-specific factors were used in the second stage (40 variables for surgery, 20 for oncology, 17 for orthopedics). Model performance revealed the highest AUC in the Department of Oncology (0.879), surpassing the first-stage model (0.787). The model did not show a higher AUC in other departments, possibly due to the large sample size and overlapping risk factors. This demonstrates the advantages of machine learning in broadening the opportunity to analyze more extensive parameters beyond the classic risk factors incorporated in the traditional scoring systems. However, prior to clinical implementation, these AI driven assessment tools must be evaluated and validated in clinical trials to ensure reliability and accuracy.

### 5.5. AI-Based Guidance for Anticoagulation Approach

Determining in which patients to initiate prophylactic anticoagulation and the duration of this can be challenging. To study the utility of AI in assisting with these decisions, Park et al. conducted a retrospective study analyzing 158,804 EHRs and created prediction models incorporating patient demographics, ICD codes, medications, and length of hospital stay [43]. They were able to successfully identify patients at risk for PE post-discharge, with the best model having an AUC of 0.84, sensitivity of 0.74, and specificity of 0.80. This information could enable targeted prophylactic anticoagulation to reduce risk of PE in high-risk patients, avoiding potential complications in lower-risk patients.

The premature discontinuation of anticoagulation therapy within 90 days due to major bleeding risk can lead to severe consequences, such as recurrent PE. Mora et al. utilized machine learning on data from the Registro Informatizado de Enfermedad TromboEmbólica (RIETE) registry to create a model with numerous variables to predict patients at risk for recurrent PE or fatal PE 30 days after discontinuation of treatment (AUC 0.96, 95% CI: 0.95–0.98) [23]. Utilizing such a model on a larger scale can potentially guide clinicians in selecting an appropriate duration of anticoagulation.

### 5.6. PE Detection by AI in Post-Surgical Patient Populations

Surgery is an independent and major risk factor for developing PE. A recent study developed an ML model to specifically assess postoperative PE risk after hernia repair [44]. The model was developed using data from 2856 patients in the CHAT-1 clinical trial [45]. Eleven preoperative and fifteen combined variables from the Caprini and Padua risk assessment models were utilized to develop and test nine different ML approaches [46,47]. On average, all models had an AUC > 0.60. The TabNet ML algorithm achieved the highest AUC at >0.65. The model identified several important predictors of post-hernia repair PE, including prior personal or family history of PE, use of oral contraceptives or hormone replacement, interruption of anticoagulant therapy, open surgical technique, reducible hernia, use of general anesthesia, and compressed venous recovery time less than 24 h. Many of these are surgery-specific risk factors. In a similar study by Shohat et al., they developed ML models to predict PE after total knee arthroplasty (TKA) in a population of 35,963 TKA patients, including 308 PE cases (170 PEs), using four ML models. The gradient-boosting tree model achieved the highest AUC for both outcomes (PE: 0.774 [SD 0.055]) [48].

## 6. Current Limitations and Future Directions

The field of AI has rapidly gained the spotlight and has shown potential in being incorporated into diagnostic and therapeutic tools for PE. Studies have demonstrated that AI can potentially help with multiple aspects of care, including PE risk assessment, PE identification on imaging, and assistance with clinical decision-making regarding treatment. However, the general reproducibility of these findings is limited by small-scale studies in limited populations. Future research can overcome this by using larger, more robust clinical trials which can work on standardization of data and creating universally adaptable algorithms.

NLP may be incorporated directly into the EHR, rapidly performing a PE risk assessment for all admitted patients, with a warning for those at a particularly increased risk of bleeding or clotting. This can assist in decision-making regarding imaging requests and prophylactic treatment, using extensive patient factors that may not be immediately identified by clinicians. The result of this is the avoidance of unnecessary testing or medication use, cutting down the associated potential adverse events. However, this is currently limited by the non-standardized documentation in EHRs. Until it is seamlessly integrated into most EHRs, using AI will be an extra step that many clinicians would hesitate to utilize.

Beyond this, AI through CNNs may then be incorporated into radiology systems, with automatic assessment of scans, highlighting areas of suspicion for radiologist review. This would reduce time to diagnosis and increase diagnostic accuracy and sensitivity. In patients with PE, treatment decisions can be guided by AI risk assessment of factors such as potential PE recurrence and bleeding risk. Outside of immediate clinical practice, CNNs may be used to identify new imaging indicators for prediction of PE prognosis.

AI has the potential to address many clinical challenges by facilitating large-scale data analysis and offering insights that can lead to more precise and personalized tools. This seamless integration would naturally fit with modern extensive EHR systems but would require extensive infrastructure development that may yet be unavailable in many clinical contexts. It would also necessitate further development of AI, with improvement in accuracy, sensitivity, and specificity. As the field evolves, continued interdisciplinary collaboration will be crucial to harnessing the full potential of AI in PE diagnosis and management.

However, a limitation, which applies to any system change, is that it requires training and modification of already established habits and workflows, which might limit its acceptance.

## 7. Conclusions

Artificial intelligence is a powerful tool capable of analyzing vast amounts of data from imaging, electronic health records, and hospital systems, enabling early detection of pulmonary embolism and optimizing patient care. Machine learning algorithms have demonstrated potential in accurately predicting pulmonary embolism risk and determining the appropriate duration of anticoagulation. Additionally, convolutional neural networks may assist in enhancing the speed and diagnostic accuracy of radiologists in reading CT pulmonary angiographies. Further studies involving larger cohorts are needed to validate these findings, which can potentially improve patient safety and facilitate timely care.

## Figures and Tables

**Table 1:** Studies evaluating the FDA-approved AI model from Aidoc© Medical (external validation).

Author and Year	AI Model	Details of Population	Number of CT Scans	Diagnostic Performance
Buls, et al. [16]	AIDOC Version 1.3	All consecutive CTPA scans performed between 1 July 2019 and 1 February 2020 for any reason	448 CTPAs	Sensitivity: 73% Specificity: 95% PPV: 73% NPV: 94%
Cheikh, et al. [17]	AIDOC version 1.0	All consecutive adults with suspected PE obtaining CTPA between 21 September 2019 and 24 December 2019	1202 CTPAs	Sensitivity: 92.6% Specificity: 95.8% PPV: 80.4% NPV: 98.6%
Langius-Wiffen, et al. [18]	AIDOC (version not specified)	All consecutive adults with suspected PE obtaining CTPA between 24 February 2018 and 31 December 2020	3316 CTPAs	Sensitivity: 96.8% Specificity: 99.9% PPV: 99.7% NPV: 99.1%
Zaazoue, et al. [19]	AIDOC (version not specified)	Hospitalized adult COVID-19 patients receiving contrast enhanced chest CTs. All scans positive for PE (527) and selected matched controls (977) were included	1504 contrast-enhanced CT cans	Sensitivity: 93.2% Specificity: 99.6%

## Data Availability

The data that support the findings of this study are available in this article, and further inquiries can be directed to the corresponding author.
